# Dr. Walsh's Proposed Laboratory Clinic

**Published:** 1913-12-20

**Authors:** Edwin L. Ash

**Affiliations:** London, W.


*To the Editor of* The Hospital.

### Dr. Walsh's Proposed Laboratory Clinic.

*To the Editor of* The Hospital.

Sir,—Whilst Dr. Walsh's scheme for the establishment of a central public laboratory clinic is in essence a sound plan for the better organisation of the treatment and diagnosis of several diseases which should now be dealt with in the clinical laboratory rather than in the ordinary consulting-room, it surely errs through over-zeal on the part of its promoter. In the first place, the scheme as outlined in your issue of December 13 suggests the establishment of an institute on very elaborate lines; and, indeed, so far as I can make out, nothing less than the foundation of a new special hospital with modern bacteriological laboratories would fit the given suggestions.

But one would have thought that a moderate-sized suite of rooms under the control of a couple of experienced men would be sufficient to put into practice the excellent idea which the scheme embodies—namely, that of a central laboratory where persons of small means, as well as the indigent sick, could be seen by their doctors for the purposes of the various elaborate diagnostic and therapeutic measures which modern medical science has decided must be used in many cases.

Again, the superintendent of the laboratory clinic should surely devote his whole time to the work and not be a "part-time" officer, as suggested in paragraph 3 of "The Scheme in Outline." Further, as to the method of upkeep, it is far better that all schemes of this kind should be based on a co-operative plan, such that the various sections of the public who will derive benefit from the institution shall pay in proportion to their means, and on such a scale that, whilst it can treat the very poor for nothing, the fees derived from persons who can pay will suffice to meet the necessary expenses.

Personally I should like to see clinics founded to meet the requirements of various special branches of medicine, which would provide not only for the very poor and moderately well off, but where the well-to-do might obtain the advantages offered by the payment of substantial fees, which, together with payments derived from an in-patient department, would suffice to keep things going without any appeal to charity.—Faithfully yours,

Edwin L. Ash.

London, W., December 16, 1913.

